# Associations of Gut Microbiota With Heat Stress-Induced Changes of Growth, Fat Deposition, Intestinal Morphology, and Antioxidant Capacity in Ducks

**DOI:** 10.3389/fmicb.2019.00903

**Published:** 2019-04-26

**Authors:** Jun He, Yuxin He, Daodong Pan, Jinxuan Cao, Yangying Sun, Xiaoqun Zeng

**Affiliations:** ^1^Key Laboratory of Animal Protein Deep Processing Technology of Zhejiang Province, Ningbo University, Ningbo, China; ^2^Department of Food Science and Nutrition, Nanjing Normal University, Nanjing, China

**Keywords:** gut microbiota, heat stress, intestinal injury, antioxidant capacity, 16S rRNA, Cherry-Valley duck

## Abstract

Accumulating evidence has revealed the dysbiosis of gut/fecal microbiota induced by heat stress (HS) in mammals and poultry. However, the effects of HS on microbiota communities in different intestinal segments of Cherry-Valley ducks (a widely used meat-type breed) and their potential associations with growth performances, fat deposition, intestinal morphology, and antioxidant capacity have not been well evaluated yet. In this study, room temperature (RT) of 25°C was considered as control, and RT at 32°C for 8 h per day was set as the HS treatment. After 3 weeks, the intestinal contents of jejunum, ileum, and cecum were harvested to investigate the microbiota composition variations by 16S ribosomal RNA amplicon sequencing. And the weight gain, adipose indices, intestinal morphology, and a certain number of serum biochemical parameters were also measured and analyzed. The results showed the microbial species at different levels differentially enriched in duck jejunum and cecum under HS, while no significant data were observed in ileum. HS also caused the intestinal morphological changes (villus height and the ratio of villus height to crypt depth) and the reductions of growth speed (daily gain), levels of serum triglyceride (TG) and total cholesterol, and antioxidant activity (higher malondialdehyde (MDA) content and lower total antioxidant). The higher abdominal fat content and serum glucose level were also observed in HS ducks. The Spearman correlation analysis indicated that in jejunum the phyla *Firmicutes* and *Proteobacteria* were associated with average daily gain, feed/gain, serum TG and MDA levels, and villus height/crypt depth (*P* < 0.05). The phylum *Firmicutes* and genus *Acinetobacter* were significantly associated with fat deposition and serum glucose level (*P* < 0.05). The genus *Lactobacillus* was positively associated with serum total antioxidant (*P* < 0.05), while some other microbial species were found negatively associated, including order *Pseudomonadales*, genera *Acinetobacter*, and *unidentified_Mitochondria*. However, no significant correlations were observed in cecum. These findings imply the potential roles of duck gut microbiota in the intestinal injuries, fat deposition, and reductions of growth speed and antioxidant capacity caused by HS, although the molecular mechanisms requires further investigation.

## Introduction

Heat stress (HS) is a great threat to livestock industries, especially in the past decades due to the global warming and has induced major economic loss (St-Pierre et al., 2003; Hansen et al., 2010; Mack et al., 2013; Nawab et al., 2018). HS has a profound effect on animal health and production performance (Humphrey, 2006). HS causes multiple physiological disturbances, such as endocrine disorders, immune dysregulation, electrolyte imbalance, and so on (Teeter et al., 1985; Sohail et al., 2010). And also, HS could lead to the abnormalities of energy metabolism, fat deposition, and body oxidative status (Baziz et al., 1996; Küchenmeister et al., 1999; Rhoads et al., 2009; Faylon et al., 2015; He et al., 2018).

In recent years, gut microbiota, considered as a “microbial organ,” has become a central research focus because it has a symbiotic relationship with host health and plays an essential role in the nutrient digestion and absorption, immune system development, and host protection against pathogens (Leser and Molbak, 2009; Vasai et al., 2014b; Whiteside et al., 2015). Balanced intestinal microflora benefit host by excluding pathogens, improving intestinal barrier integrity, maintaining normal nutrient digestion and absorption, promoting other commensals, and so on (Burkholder et al., 2008; Song et al., 2013). However, it has been reported that HS negatively affects intestinal mucosa and microbiota composition (Liu et al., 2009; Kers et al., 2018). Damage to mucosal epithelium can directly affect intestinal barrier function, nutrient absorption and impair production performance (Elphick, 2005; Moeser et al., 2007). With inefficient heat dissipation and lack of sweat glands, poultry appears more susceptible to HS (Lara and Rostagno, 2013). In chicken, some reports are available about the influences of HS on gut microbiota in different intestinal segments and the use of dietary supplementations to improve intestinal morphology and microflora balance, like oligosaccharide (Song et al., 2013; Ghasemian and Jahanian, 2016), *N*-acetylcysteine (Yi et al., 2016), *Artemisia annua* (Song et al., 2017), and probiotics (Al-Fataftah and Abdelqader, 2014; Song et al., 2014). The data about gut microbiota in different duck breeds, including Peking, Muscovy, Sheldrake and Mule, are mainly on the application of overfeeding or functional dietary supplementation to promote production performances (Jiang et al., 2014; Vasai et al., 2014a,b). Similar data could also be found on zebrafish (Wang et al., 2019). Whereas, the effects of HS on the gut microbiota composition and its association with physiological changes have not been well evaluated.

In the present study, Cherry-Valley duck, a widely used meat-type breed, was selected to investigate the influences of HS on duck gut microbiota. After thermal treatment, the 16S rDNA sequencing analysis was performed to learn the variations of microflora compositions in different intestinal segments (jejunum, ileum, and cecum), and probe into their possible associations intestinal morphology, fat deposition, and oxidation status. The results help better understand the effects of HS on duck physiology and the probably involved bacterial species, and it might provide useful information for preventing or ameliorating the deficits of duck production caused by prolonged high-temperature environment.

## Materials and Methods

### Animal Treatment and Sampling

A total of 30 Cherry-Valley ducks (male, 5-week-old, 1.5∼1.8 kg) with the same genetic background were collected and randomly divided into 2 groups, control (C), and treatment (T). Each duck was housed in a separate cage. In the first week, the room temperature (RT) was controlled at 25°C for adaption. Then for the next 3 weeks, the RT of group T was promoted to 32°C and kept for 8 h per day (10:00 am to 18:00 pm), while no RT change was made to group C. Throughout the whole experimental period, all birds were watered and fed *ad libitum*. The diet ingredients and nutrient contents are listed in Table 1. After fasting for 12 h, the birds were weighed and the blood samples were harvested. Then 5 individuals of each group were randomly selected and euthanatized by jugular venesection. The intestinal contents of jejunum, ileum and cecum carefully sampled, immediately frozen into liquid nitrogen and lately stored at −70°C. The abdominal fat tissue was isolated and weighed. The content of abdominal fat was calculated as its proportion of the eviscerated carcass weight. Meanwhile, for each duck, a 2-cm section of each intestinal segment and a piece of abdominal fat tissue were separated and submersed in 10% neutral-buffered formalin for 24 h of fixation.

**Table 1:** Ingredients and nutrient composition of the diet for the experimental ducks.

Ingredients (%)	
Corn	64.9
Wheat bran	6.35
Soybean meal	18
Cottonseed cake	4
Fish meal	2
Soy oil	1.5
Limestone	0.9
Calcium bicarbonate	1.1
Salt	0.25
Premix	1
Total	100
Nutrient contents	
Metabolizable energy (MJ/kg)	12.19
Crude protein	17.16
Calcium	0.86
Phosphorus	0.48
Lysine	0.80
Methionine + cystine	0.65

*Premix provided for each kg of complete diet: VA, 10000 IU; VD_3_, 1000 IU; VE, 20 IU; VK_3_, 2 mg; VB_1_, 2 mg; VB_2_, 5 mg; VB_6_, 2 mg; folic acid, 0.6 mg; nicotinic acid, 30 mg; pantothenic acid, 11 mg; biotin, 0.2 mg; Fe, 70 mg; Zn, 50 mg; Cu, 8 mg; I, 0.4 mg; and Se, 0.2 mg.*

### DNA Extraction and 16S rRNA Amplicon Sequencing

Total genomic DNA of the sampled intestinal contents was extracted using QIAamp DNA isolation kit (Qiagen, Hilden, Germany). The concentration and integrity of bacterial DNA were assessed with a Nanodrop (Thermo Fisher Scientific, United States) and 1.5% agarose gel electrophoresis, respectively. The diluted DNA (1.0 ng/μL) was used to amplify the V4 hypervariable regions the 16S rRNA gene amplicons with the barcoded primers (515F, 5′-GTGCCAGCMGCCGCGGTAA-3′;806R, 5′-GGACTACHVGGGTWTCTAAT-3′) and Phusion^®^ High-Fidelity PCR Master Mix with GC Buffer (New England Biolabs). The PCR products were subjected to 2.0% agarose gel electrophoresis, recovered, and purified using GeneJET Gel Extraction Kit (Thermo Fisher Scientific, United States). Sequencing libraries were generated using Ion Plus Fragment Library Kit (Thermo Fisher Scientific, United States) according to the manufacturer’s recommendations. Library quality was assessed on the Qubit*@* 2.0 Fluorometer (Thermo Fisher Scientific, United States) and Agilent Bioanalyzer 2100 system. Finally, the library was sequenced on Ion S5^TM^ XL platform (Thermo Fisher Scientific, United States).

### Microbial Bioinformatic Analysis

The low-quality reads of the original data were filtered with Cutadapt (V1.9.1) (Martin, 2011), and the data of each sample was separated by barcode. Then the barcode and primer sequences were cut off to obtain the Raw Reads, which further went through alignment with Gold database^1^ (Haas et al., 2011) using UCHIME Algorithm (Edgar et al., 2011) to remove the chimeric sequences and turned out the Clean Reads. The Clean Reads were then clustered as operational taxonomic units (OTUs) by scripts of Uparse software (version 7.0.1001) with a 97% similarity threshold.

The representative OTUs (high frequency) were screened and annotated with SILVA SSUrRNA database using Mothur (threshold 0.8∼1.0) (Wang et al., 2007; Quast et al., 2012). Then MUSCLE software (version 3.8.31) was applied to perform the multiple sequence alignment of the OTUs to generate their phylogenetic relationship. And the OTU abundances were normalized using a standard of sequence numbers corresponding to the sample with the least sequences, based on which the diversity analysis were performed. To estimate of the microbial community of the samples, the within-sample alpha-diversity was calculated according to the genera profiles. Beta-diversity was estimated by calculating Unweighted-Unifrac and Weighted-Unifrac distances, then visualized with principal coordinate analysis (PCoA) and non-metric multi-dimensional scaling (NMDS). Kyoto Encyclopedia of Genes and Genomes (KEGG) pathway enrichment analysis was performed to predict the microbial functions. Linear discrimination analysis coupled with effect size (LEfSe) was performed to identify the bacterial taxa differentially represented between/among groups at different taxonomy levels (Segata et al., 2011). The Spearman correlation analysis was applied to analyze the associations of the differential microbial species with the measured parameters.

### Preparation and Analysis of Intestinal and Adipose Tissue Sections

According reported methods (Murugesan et al., 2015; Varlamov et al., 2017), after fixation and dehydration, samples of different intestinal segments and abdominal tissue were embedded (EG1150H, LEIC, Germany) in paraffin wax, sliced (rotary microtome, RM2016, LEIC, Germany) and stained with hematoxylin and eosin in preparation for examination by microscope (S4E, LEIC, Germany), and image analyzer (Image-ProPlus 6.0). For each intestinal sample, 5 villus were selected for Villus height (VH), crypt depth (CD), mucosal thickness determination. For each adipose section, at least 3 vision fields were chosen and for each a certain range (containing 20–50 adipocytes) was circled out. Then the area and number of each range was measured with the image analyzer to calculate the average area and density of adipocytes.

### Determination of Serum Biochemical Parameters

The collected blood samples were firstly incubated at RT for 1∼2 h, centrifuged at 3,000 rpm, 4°C for 15 min to obtain serum, and then stored at −80°C until analysis. The energy metabolism and oxidative status related parameters of the collected serum samples were determined, including levels of glucose (GLU), triglyceride (TG), high density lipoprotein-cholesterol (HDL-C), low density lipoprotein-cholesterol (LDL-C), total cholesterol (T-CHO), malondialdehyde (MDA), superoxide dismutase (SOD), and total antioxidant capacity (T-AOC). All the determinations were performed on a Multifunctional Microplate Reader (Infinite^®^ M200PRO, TECAN, Switzerland) with related commercial reagent kits (Jiancheng, Nanjing, China) according to the manufacturer’s recommendations. Index measurements of each sample were replicated 3 times.

### Statistical Analysis

Body weight, serum biochemical parameters, intestinal and adipose traits were presented as mean ± standard deviation (SD). *T*-test was applied with SPSS software (Version 20.0) to analyze the differences between group C and T. The results were considered significantly different at *P* < 0.05.

## Results

### Effects of HS on Body Weight and Fat Deposition

**FIGURE 1:**
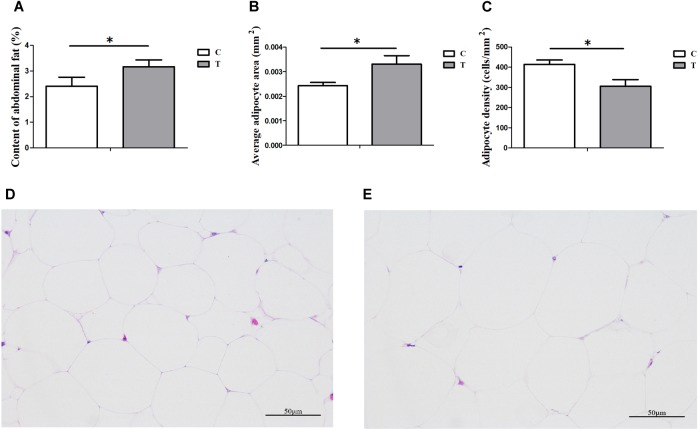
Influences of heat stress (HS) on abdominal fat deposition in duck (*n* = 5 for each group). C and T stand for control and thermal treatment, respectively. The abdominal fat content **(A)**, average adipocyte area **(B)**, and cell density **(C)** were calculated, and the statistical differences between C and T were analyzed (^∗^*P* < 0.05). The adipose tissue sections (400×) of C **(D)** and T **(E)** were also presented here.

Under HS for 3 weeks, the average daily gain of Cherry-Valley ducks were measured significantly less than control (*P* = 0.0098), with a higher Feed/Gain ratio (*P* = 0.0467) (Table 2). However, the abdominal fat content significantly increased in T, compared to C (*P* = 0.0396) (Figure 1A). And the morphology results of abdominal fat tissue also showed higher single adipocyte area (*P* = 0.0321) and lower cell density (*P* = 0.0240) in thermal-treated birds (Figure 1B,C).

**Table 2:** Effects of heat stress (HS) on growth performance of Cherry-Valley ducks.

Items	Control	HS	*P* values
Initial weights (kg)	1.643 ± 0.092	1.622 ± 0.076	0.8103
Final weights (kg)	4.203 ± 0.156	3.467 ± 0.174	0.0105
ADG (g)	91.43 ± 2.45	65.89 ± 5.25	0.0098
Feed/Gain	2.65 ± 0.12	3.01 ± 0.13	0.0467

*Dada are expressed as mean ± SD. ADG, average daily gain; Feed/Gain, the ratio of feed intake to body weight gain.*

### Morphological Damages to Different Intestinal Segments When Exposed to HS

**FIGURE 2:**
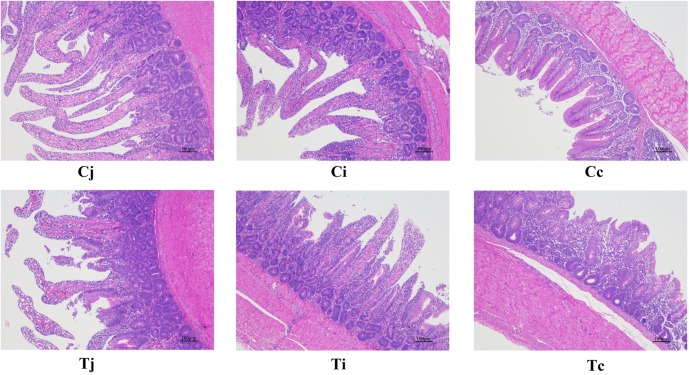
Tissue sections (100×) of intestinal mucosa in jejunum, ileum, and cecum in ducks with/without HS (*n* = 5 for each group). **Cj**, **Ci**, and **Cc** stand for the jejunum, ileum, and cecum of control. **Tj**, **Ti**, and **Tc** stand for the jejunum, ileum, and cecum of the thermal treated group, respectively. This also applies to the following figures.

For both groups, the morphological changes of jejunum, ileum, and cecum were presented in Table 3 and Figure 2. It showed that VHs in jejunum and ileum were both shorter in T, compared with C (*P* < 0.05). No significant differences of the CDs in all the three intestinal segments were found (*P* > 0.05). Then the VH/CD values were calculated higher in both jejunum and ileum of C (*P* < 0.05).

**Table 3:** Morphological changes of jejunum, ileum and cecum in ducks under heat stress (HS).

Intestinal segments		Control	HS	*P* values
Jejunum	VH (mm)	0.699 ± 0.042	0.558 ± 0.067	0.0100
	CD (mm)	0.128 ± 0.029	0.143 ± 0.014	0.3704
	VH/CD	5.734 ± 1.235	3.935 ± 0.612	0.0411
Ileum	VH (mm)	0.528 ± 0.024	0.348 ± 0.023	<0.0001
	CD (mm)	0.087 ± 0.094	0.094 ± 0.017	0.5619
	VH/CD	6.294 ± 1.280	3.895 ± 1.084	0.0217
Cecum	CD (mm)	0.272 ± 0.030	0.252 ± 0.042	0.3776

*Dada are expressed as mean ± SD. VH, villus height; CD, crypt depth.*

### Serum Biochemical Variation After Heat Treatment

The levels of serum Glu, TG, HDL-C, LDL-C, T-CHO, MDA, SOD, and T-AOC were determined and listed in Table 4. It indicated that compared to group C, the level of Glu was significantly higher (*P* = 0.0228), while the levels of TG, T-CHO, MDA, and T-AOC were all found significantly lower (*P* < 0.05) in group T. And no significant difference was detected on HDL-C, LDL-C or SOD between C and T (*P* > 0.05).

**Table 4:** Effects of heat stress (HS) on serum indexes in cherry-valley duck.

Serum parameters	Control	HS	*P* values
Glu (mM)	7.515 ± 0.310	8.461 ± 0.532	0.0228
TG (mM)	1.105 ± 0.276	0.717 ± 0.101	0.0458
HDL-C (mM)	6.291 ± 0.714	5.046 ± 0.692	0.0831
LDL-C (mM)	0.357 ± 0.072	0.273 ± 0.043	0.0943
T-CHO (mM)	5.497 ± 0.731	3.983 ± 0.458	0.0444
MDA (μM)	7.347 ± 0.503	9.067 ± 0.646	0.0062
SOD (U/mL)	20.068 ± 0.574	20.900 ± 0.708	0.1554
T-AOC (=mM FeSO_4_)	1.325 ± 0.261	0.777 ± 0.106	0.0153

*Dada represent as mean ± SD. Levels of Glu, glucose; TG, triglyceride; HDL-C, high density lipoprotein-cholesterol; LDL-C, low density lipoprotein-cholesterol; T-CHO, total cholesterol; MDA, malondialdehyde; SOD, superoxide dismutase; and T-AOC, total antioxidant in the groups of control and HS are shown here, respectively. The T-AOC levels represent the equivalent antioxidant capacity of FeSO_4_.*

### Microbiota Compositions in the Sampled Intestinal Segments

**FIGURE 3:**
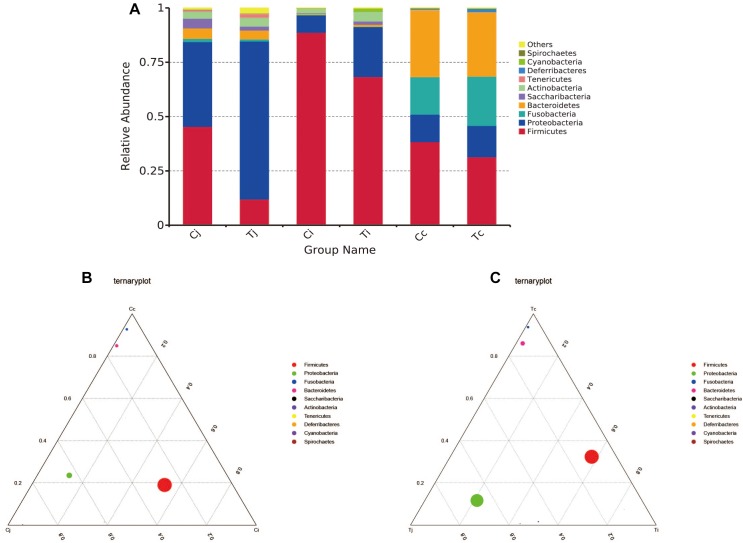
Microbiota compositions in different intestinal segments of ducks with/without HS (*n* = 5 for each group). Top 10 bacterial phyla in jejunum, ileum and cecum of the used Cherry-Valley ducks in this study **(A)**. And the ternary analysis of the 3 intestinal positions in group C and T are shown in **(B**,**C)**, respectively.

**FIGURE 4:**
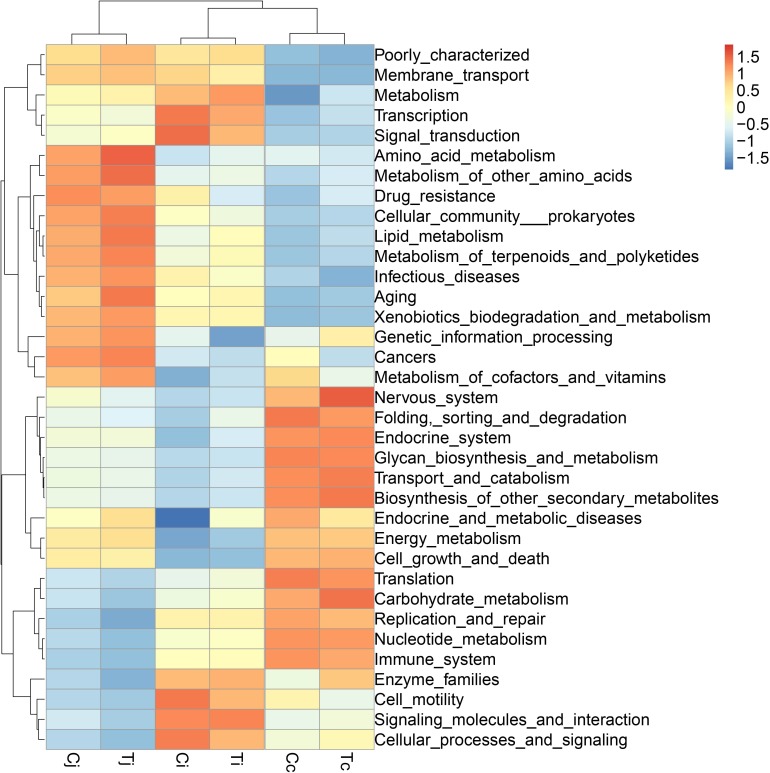
The KEGG pathway enrichment analysis of the gut microbiota in ducks. The different colors represent the distance between the raw score and the mean population of the standard deviation.

By 16S rRNA gene sequencing, the gut microbiota composition at phylum level of C (Cj, Cj, and Cc) and T (Tj, Ti, and Tc) was calculated (Figure 3A) and the dominant species were analyzed and shown in ternary plot (Figure 3B,C). It indicated that *Firmicutes* and *Proteobacteria* were major microbiota communities in Cj (mean, 84.5%), Ci (mean, 96.6%), Tj (mean, 84.7%), and Ti (mean, 91.3%). *Firmicutes*, *Proteobacteria*, *Fusobacteria*, and *Bacteroidetes* altogether comprised 98.0 and 98.9% of microbiota in Cc and Tc, respectively. Top 10 microbiota relative abundances at levels of class, order, family, and genus were also analyzed and presented in Supplementary Figure S1. The shared and specific OTUs in the two groups (Supplementary Figures S2A,B) and in the 3 segments with different treatments (Supplementary Figures S2C–E) were also calculated. The result of KEGG pathway analysis was listed in Figure 4.

### Alpha and Beta Diversity of Gut Microbiota Affected by Heat Stress

**FIGURE 5:**
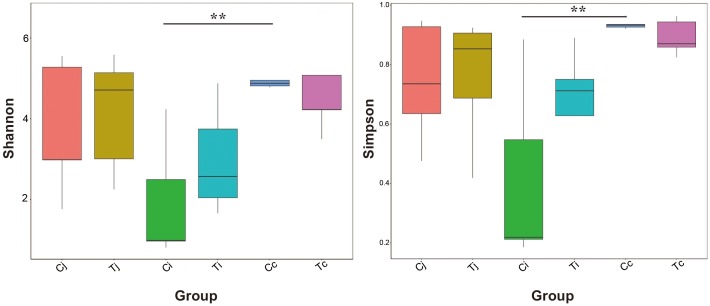
Bacterial species richness and diversity in the 3 intestinal segments of group C and T. The Shannon index and Simpson index were used to assess the species richness and diversity (^∗∗^*P* < 0.01).

**FIGURE 6:**
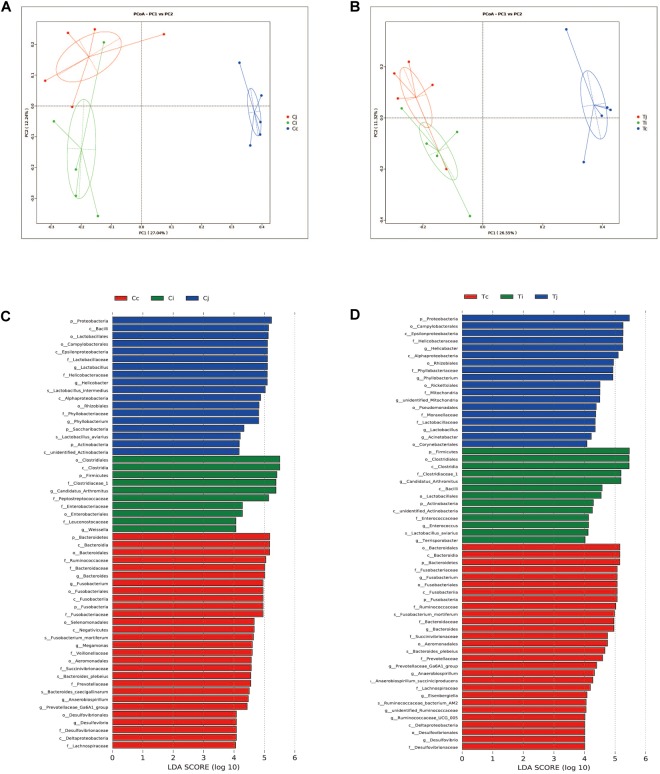
Differential analysis of microbiota community among the 3 intestinal segments within group C and T. The principal coordinate analysis (PCoA) based on Binary-Jaccard distance was applied to observe the separation of the samples from C **(A)** and T **(B)**. The Linear discrimination analysis (LDA) coupled with effect size (LEfSe) was used to identify the most differentially abundant taxa among the 3 intestinal segments within group C **(C)** and T **(D)**. Only the results meeting an LDA significant threshold of >4 were shown.

**FIGURE 7:**
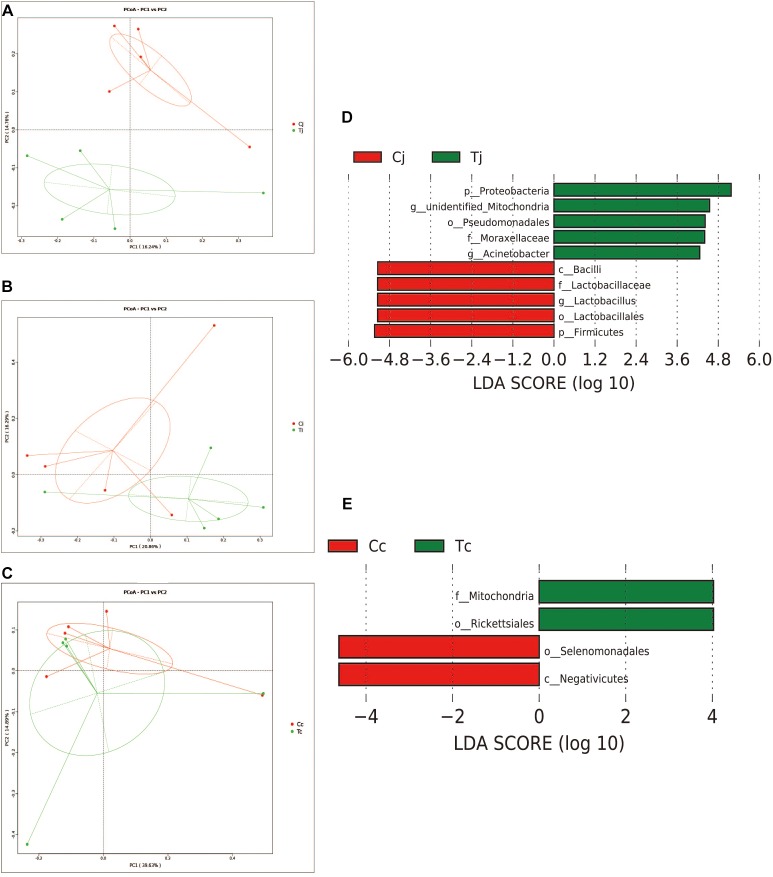
Effects of HS on the microbiota compositions in the 3 intestinal segments. The Binary-Jaccard based PCoA analysis revealed the different effects of HS on the samples from jejunum **(A)**, ileum **(B)**, and cecum **(C)**. The LEfSe analysis identified the differentially abundant (LDA score >4) bacterial species induced by HS in jejunum **(D)** and cecum **(E)**, but not in ileum.

Two indexes that reflecting species richness and diversity (Shannon and Simpson) decreased in Ci compared with Cc (*P* < 0.01) (Figure 5), while no significant difference was found between any other two segments in C or T. The Binary-Jaccard based PCoA results showed that the cecal microbiota was separated clearly from that in jejunum or ileum of both C and T (Adonis analysis, *P* < 0.05), and for both the 2 groups, the jejunum microbiota was well separated from that in ileum (Adonis analysis, *P* < 0.05) except for one sample spot (Figure 6A,B). To identify bacterial taxa that significantly differentiated among jejunum, ileum and cecum, a metagenomic biomarker discovery approach (LEfSe) was applied (LDA score >4) and the results were listed in Figure 6C,D. Moreover, The Binary-Jaccard based PCoA also showed that Cj displayed a distinct microbiota community that clustered separately from Tj (Adonis analysis, *P* < 0.05) (Figure 7A), while neither Ci-Ti nor Cc-Tc were separated into different clusters (Adonis analysis, *P* > 0.05) (Figure 7B,C). The NMDS analysis showed similar results to PCoA (Supplementary Figure S3). By LEfSe analysis, the differentially enriched microbiota (LDA score >4) at different classification levels were found in jejunum and cecum. *Firmicutes* (phylum), *Bacilli* (class), *Lactobacillales* (order), *Lactobacillaceae* (family), and *Lactobacillus* (genus) were more abundant in Cj. *Proteobacteria* (phylum), *Pseudomonadales* (order), *Moraxellaceae* (family), *Acinetobacter* (genus), and an unclassified member of *Mitochondria* (genus) were significantly enriched in Tj (Figure 7D). The relative abundances of *Rickettsiales* (order) and *Mitochondria* (family) markedly increased in Tc, while *Negativicutes* (class), and *Selenomonadales* (order) were more prevalent in Cc (Figure 7E). However, no significant differences of microbiota abundance were detected between Ci and Ti.

### Correlation Between the Differential Microbial Species and Measured Parameters

**FIGURE 8:**
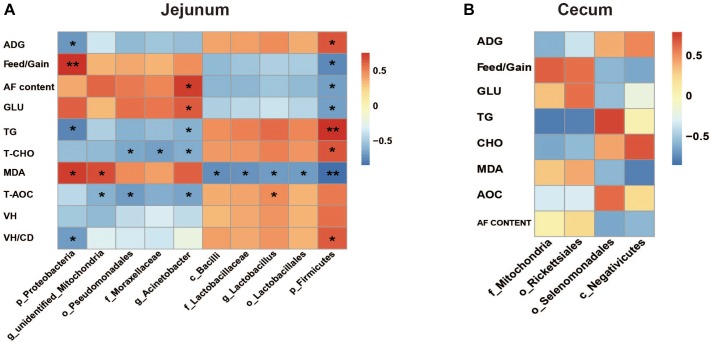
The Spearman correlation analysis between the differential microbial species and measured parameters in jejunum **(A)** and cecum **(B)**. ADG, average daily gain; AF, abdominal fat; Glu, glucose; TG, triglyceride; T-CHO, total cholesterol; MDA, malondialdehyde; T-AOC, total antioxidant; VH, villus height; CD, crypt depth. The red represents positive correlation and the blue represents negative correlation, respectively (^∗^*P* < 0.05, ^∗∗^*P* < 0.01).

The Spearman correlation analysis results were presented in Figure 8. The results in jejunum showed that ADG and VH/CD were associated with phyla *Firmicutes* (positive, *P* < 0.05) and *Proteobacteria* (negative, *P* < 0.05), and Feed/Gain represented the opposite associations (*P* < 0.05). Abdominal fat content and serum GLU level were positively associated with genus *Acinetobacter* and negatively associated with phylum *Firmicutes* (*P* < 0.05). TG and T-CHO levels represented the opposite associations to GLU and abdominal fat content, and also negatively associated with phylum *Proteobacteria*, order *Pseudomonadales*, and family *Moraxellacese* (*P* < 0.05). Two positive associations and five negative associations were observed between serum MDA content and bacterial species (*P* < 0.05). T-AOC was positively associated with genus *Lactobacillus* (*R* = 0.47, *P* = 0.016), and negatively associated with order *Pseudomonadales* (R = −0.71, *P* = 0.022), genera *Acinetobacter* (R = −0.66, *P* = 0.039), and *unidentified_Mitochondria* (R = −0.59, *P* = 0.035). Whereas, no significant associations were found in cecum microbiota (*P* > 0.05).

## Discussion

To learn the gut microbiota community structure in Cherry-Valley duck and evaluate the effects of HS on it, a 16S rRNA sequencing analysis of the intestinal contents from jejunum, ileum, and cecum was performed in the current study. It has been reported that *Firmicutes* and *Bacteroidetes* were identified as the dominant phyla in avian gut microbiota, suggesting their importance in metabolism, and host physiology (Kohl, 2012). *Firmicutes* was the major phylum in feces of Canada geese (Lu et al., 2009) and Muscovy duck (Vasai et al., 2014a), whereas phylum *Bacteroidetes* was less enriched in some avian species (Lu et al., 2007). It is consistent with the present results that *Firmicutes* was the most abundant phylum in all the 3 selected intestinal segments, jejunum (45.4%), ileum (88.7%), and cecum (38.4%). In cecum, the second abundant phylum was *Bacteroidetes* (30.6%). And together with *Fusobacteria* (17.2%) and *Proteobacteria* (12.6%), the four phyla accounted for over 98% of the cecal microbiota composition of Cherry-Valley ducks under normal feeding conditions. However, instead of *Bacteroidetes*, the second most enriched bacterial phylum turned out to be *Proteobacteria* in both jejunum (39.0%), and ileum (7.9%). At class level, *Clostridia* and *bacteroidia* (obligate anaerobes) were reported to dominate in both ileum and cecum of mule ducks (Vasai et al., 2014b). Similar data could be found in this study (Supplementary Figure S1A) except little abundance of *bacteroidia* (0.44%) in ileum. Gut microbiota is associated with nutrient digestion/absorption and host protection, and these differences might be due to different avian species/breeds or intestinal positions.

In this study, experimental animals were exposed to high ambient temperature (32°C, 8 h per day) for 3 weeks to investigate the effects of HS on gut microbiota in different intestinal segments. Although with alteration of relative abundances, the dominant bacterial phyla in ileum and cecum were found just the same as in control. In jejunum, the major phylum came out to be *Proteobacteria* (72.7%) under HS, followed by *Firmicutes* (12.0%), the second most common phylum. This compositional reshape in microbial community indicated that among the 3 intestinal segments, the most significant impacts of HS on microbiota community probably located in jejunum. HS was reported to increase intestinal permeability, decrease intestinal blood circulation and further cause damages to its integrity, which were believed to affect the colonized microbial composition (Lambert, 2009; Song et al., 2014). Some morphological changes, including VH, VH/CD or intestinal sections, were observed in all the 3 intestinal segments. However, based on LEfSe analysis, the differentially enriched bacterial at different classification levels under HS were observed in jejunum and cecum (Figure 7D,E), while no significant differences were detected in ileum. It indicated that there might be some other factors involved in the intestinal microbiota changes under HS, e.g., different segments and original microbial composition.

For example, the *Firmicutes* abundance significantly decreased in jejunum when exposed to HS. *Firmicutes* is thought to be correlated with host energy metabolism. The increase of *Firmicutes*/*Bacteroidetes* ratio was considered to be a typical characteristic of obesity-driven dysbiosis in humans and animals (Mozes et al., 2008; Cotillard et al., 2013). Here, HS caused the significant decreases of duck growth performances and serum T-CHO level. T-CHO level reflects the interaction of lipid metabolism between liver and other tissues (Heyer and Lebret, 2007). And the correlation analysis showed that the abundance of jejunum *Firmicutes* was positively associated with ADG (*R* = 0.648, *P* = 0.043) and serum T-CHO level (*R* = 0.539, *P* = 0.034). It was consistent with previous studies. On the other hand, the negative association of phylum *Proteobacteria* abundance with ADG (*R* = −0.624, *P* = 0.029) was also found. The order *selenomonadales* was reported as an acetate producer (Vargas et al., 2017) which could induce secretion of ghrelin, a “hunger hormone,” and promote food intake (Perry et al., 2016). The abundance of *selenomonadales* in cecum was observed significantly decreased under HS. But the correlation analysis suggested that its association with ADG was not significant (*R* = 0.602, *P* = 0.054). These findings still needed to be further investigated. Moreover, it was found that the serum GLU level in duck significantly increased under HS. Analogs findings have been reported in Japanese quails (Ozbey and Ozcelik, 2004). It was probably regulated by hormones to satisfy the larger body energy demand to maintain a new physiological balance, e.g., nervous system, respiratory system and fat deposition.

Heat stress was demonstrated that could promote the differentiation of adipose tissues in cattle (Rhoads et al., 2009),swine (Kouba et al., 1999), and chicken (Baziz et al., 1996). HS could increase membrane fluidity and expressions of protective proteins (Hooper and Hooper, 2005; Jiang et al., 2007; Deng et al., 2017; Zhang et al., 2018). It would further improve the insulin resistance of adipose tissue and enhance its GLU uptake to promote the TG synthesis and storage (Yu et al., 2008; O’Brien et al., 2010; Hooper et al., 2014). It was believed to be a temporary body defense mechanism to HS. The data of the abdominal fat sections showed that the adipocytes in group T appeared more differentiated than in group C, which was consistent with previous studies. The correlation analysis (Figure 8A) found the increased abundance of genus *Acinetobacter* was positively associated with the abdominal fat content (*R* = 0.729, *P* = 0.017) and abdominal fat content and serum GLU level (*R* = 0.632, *P* = 0.049), and negatively associated with the serum TG level (*R* = −0.657, *P* = 0.039), respectively. Meanwhile, the reduced abundance of phylum *Firmicutes* represented the opposite associations to *Acinetobacter*. These data indicated that jejunum microbiota might contribute to the fat deposition in duck for defending again HS, although the mechanism still needed to be further explored.

Heat stress usually induces oxidation alteration (Slawinska et al., 2019), which is closely related to intestinal barrier integrity (Song et al., 2011). And gut microbiota is believed to be closely related with gut barrier (Yang et al., 2019; Paraskeuas and Mountzouris, 2019). MDA is a product of lipid oxidation. And the serum T-AOC index was measured using FeSO_4_ as standard substance by catalyzing the reduction of Fe^3+^-TPTZ (tripyridyl-triazine) to Fe^2+^-TPTZ. In the present study, the results of MDA and T-AOC indicated the decreased antioxidative capacity in duck induced by HS. The damaged intestinal mucosa, lower VH, and VH/CD values were observed in ducks under HS. The Spearman analysis showed that the MDA content and VH/CD value were significantly associated with the abundances of jejumun *Firmicutes* and *Proteobacteria* (Figure 8A). It also showed that the abundance of *Lactobacillus* was positively associated with the T-AOC (*R* = 0.47, *P* = 0.016). It was consistent with previous reports. *Lactobacillus*, a member of the class *Bacilli* (subordinate to *Firmicutes* phylum), has been reported negatively affected by HS in broilers (Al-Fataftah and Abdelqader, 2014; Zhang et al., 2017). *Lactobacillus* was proved to be of antioxidant activity (Lin and Chang, 2000; Lee et al., 2005b), and had the capacity of scavenging free radical and reactive oxygen species (ROS) to alleviate damages induced by oxidative stress (Lee et al., 2005a; Xin et al., 2014), which could be caused by HS (Altan et al., 2003). It indicated that the reduction of genus *Lactobacillus* probably weakened the antioxidant activity of duck under HS. However, its interaction with the other 3 bacterial species (negatively associated with T-AOC) was still unclear. *Lactobacillus* was also known as amylolytic bacteria and frequently increased when fed with high-starch diets in pigs (Regmi et al., 2011). No significant association was detected between *Lactobacillus* abundance and GLU content in the present study. It was probably due to different kind of stresses or animal species.

*Lactobacillus* consists of gram-positive and facultative anaerobes that produce lactic acid, which can create low pH environment and inhibit the growth of pathogen (Rodriguez-Cabezas et al., 2010). With the damaged intestinal integrity, the less abundance of *Lactobacillus* in jejunum of group T probably enlarged the risk of pathogen amplification and invasion. For example, order *Pseudomonadales* and family *Moraxellaceae*, containing known zoonotic pathogens (George, 2005), were detected significantly increased in jejunum under HS. With respect to cecum, the microbiota compositional variation also indicated some unfavorable influences of HS. The class *negativicutes* was demonstrated negatively associated with colonitis (Warner et al., 2016), which was observed significantly decreased under HS. Moreover, the order *Rickettsiales*, which contains major species that pathogenic to animals and human (Darby et al., 2007), was also found significantly increased in HS cecum. These information indicated that more attention should be paid to the prevention of pathogen invasion in HS ducks, which has been reported in HS broilers (Alhenaky et al., 2017).

After all, there are still massive works required to be further carried out on gut microbiota involved in HS ducks. For example, the effects of the obtained associated microbial species should be carefully and respectively, validated. Under HS, the interactions between duck gut microbiota and body regulatory factors needed to be further explored and discussed, e.g., inflammatory proteins, ghrelin, peroxisome proliferators-activated receptor γ (PPARγ) or mammalian target of rapamycin (mTOR).

## Conclusion

The impacts of HS on gut microbiota community and their possible relationships with the physiological changes in Cherry-Valley ducks were investigated. The results showed that the significant microbiota compositional differences occurred in jejunum and cecum under HS, accompanied by the changes of weight gain, fat content, intestinal morphology, and oxidative indices. By Spearman correlation analysis, significant associations were found between microbiota alteration and these parameters in jejunum, but not in cecum. These data indicated that HS induced intestinal injuries, abnormal fat deposition, and reductions of growth performances and antioxidant capacity in duck, which probably have potential relationships with the gut microbiota dysbiosis.

## Ethics Statement

This study was carried out in accordance with the recommendations of the Guidelines for Experimental Animals established by the Ministry of Science and Technology (Beijing, China). The protocol was approved by the Institutional Animal Care and Use Committee of Ningbo University (Ningbo, China).

## Author Contributions

DP and JC conceived of and designed the experiments. YH performed the sampling and serum index measurement. YS wrote the tissue section. XZ and JH performed the 16S rRNA sequencing experiments. JH, DP, and JC analyzed the data. JH wrote the manuscript. All authors contributed to refining the text and approved the submitted version.

## Conflict of Interest Statement

The authors declare that the research was conducted in the absence of any commercial or financial relationships that could be construed as a potential conflict of interest.

## Footnotes

1 http://drive5.com/uchime/uchime_download.html
