# Supplementary figures and images for: Associations of Gut Microbiota With Heat Stress-Induced Changes of Growth, Fat Deposition, Intestinal Morphology, and Antioxidant Capacity in Ducks
Source: Front Microbiol. 2019 Apr 26;10:903. doi: 10.3389/fmicb.2019.00903 (PMC6498187; doi:10.3389/fmicb.2019.00903)

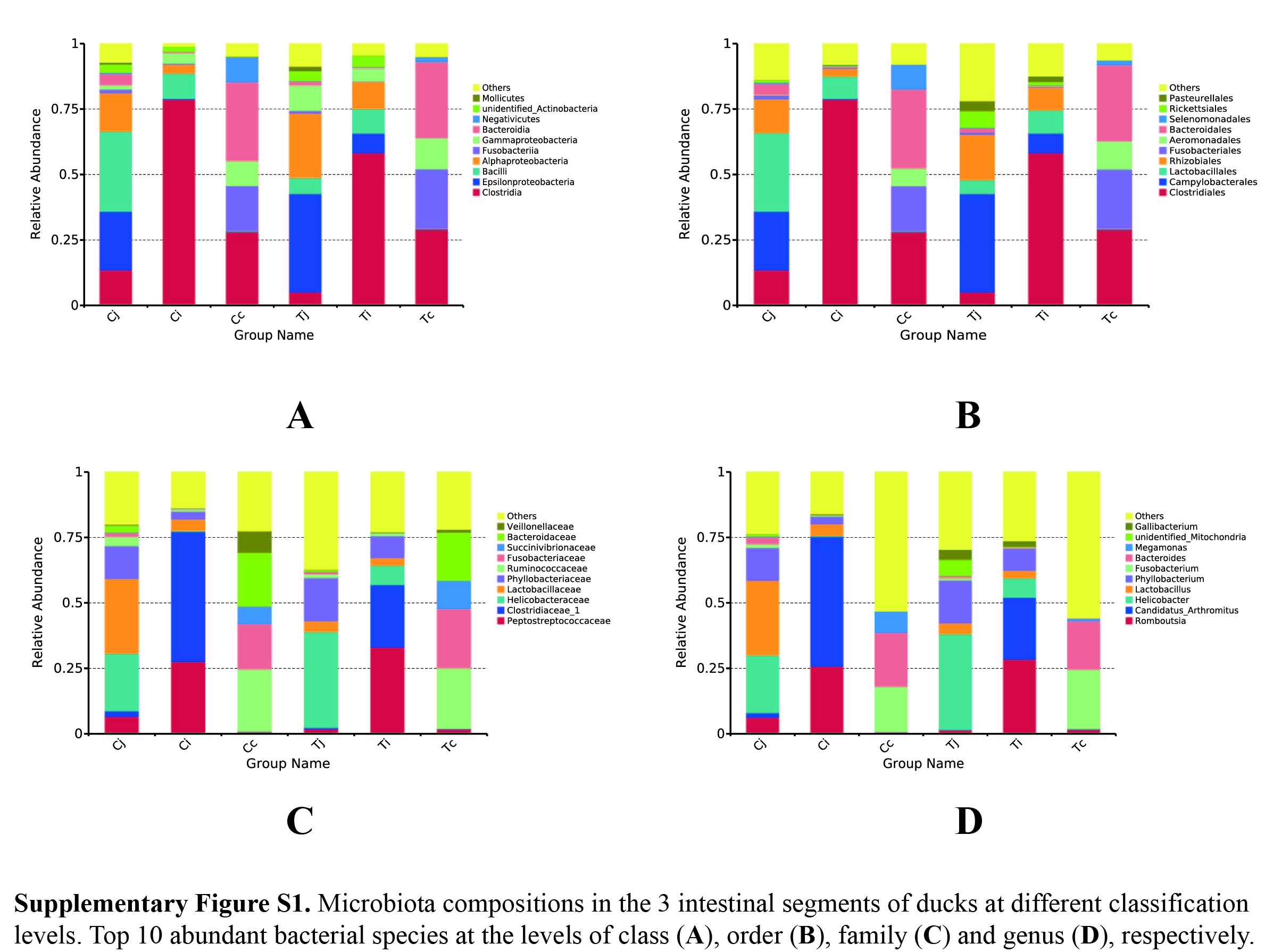

Supplement: Supplementary file 1 [file Image_1.JPEG]

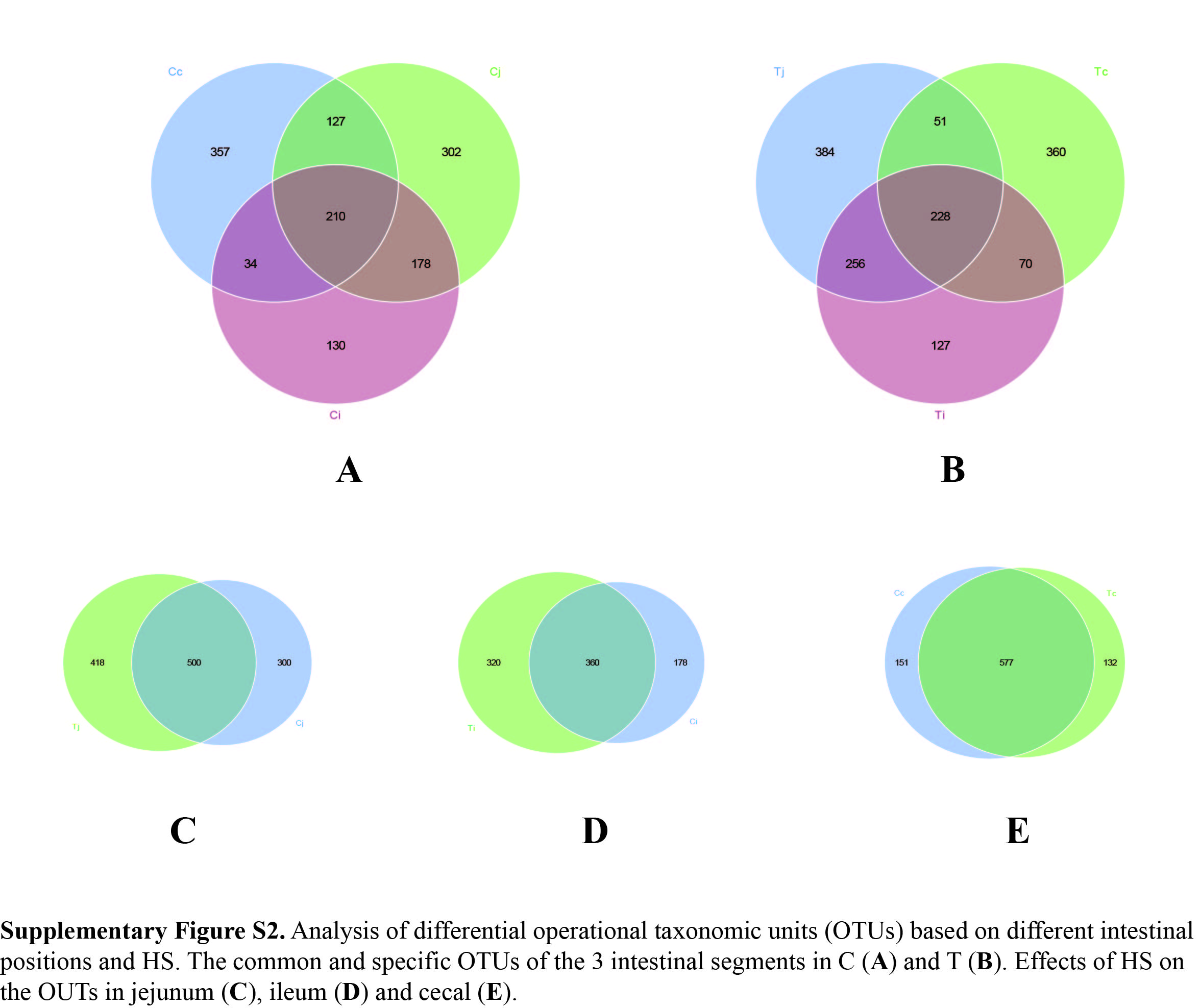

Supplement: Supplementary file 2 [file Image_2.JPEG]

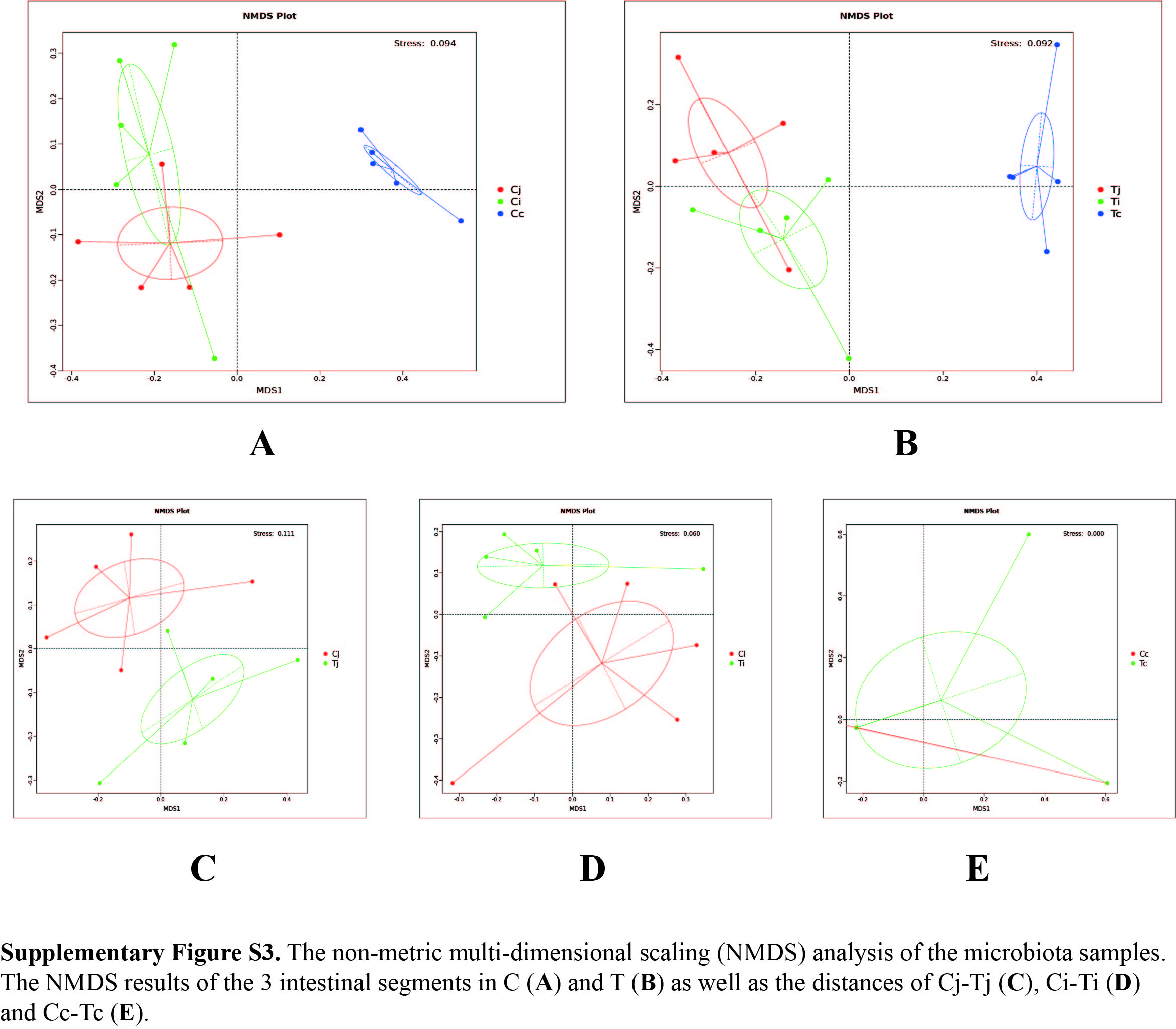

Supplement: Supplementary file 3 [file Image_3.JPEG]
